# “Clean care for all-it’s in your hands”: the May 5th, 2019 World Health Organization *SAVE LIVES: Clean Your Hands* campaign

**DOI:** 10.1186/s13756-019-0513-7

**Published:** 2019-04-17

**Authors:** Alexandra Peters, Tcheun Borzykowski, Ermira Tartari, Claire Kilpatrick, Safiah Hwai Chuen Mai, Benedetta Allegranzi, Didier Pittet

**Affiliations:** 1 Infection Control Programme and WHO Collaborating Centre on Patient Safety, University of Geneva Hospitals and Faculty of Medicine, 4 Rue Gabrielle-Perret-Gentil, 1211, 14 Geneva, Switzerland; 2 Infection Prevention and Control Global Unit, Department of Service Delivery and Safety, World Health Organization, Geneva, Switzerland

**Keywords:** Infection prevention and control, Infection control, Hand hygiene, Universal health coverage, Antimicrobial resistance, World Health Organization, Healthcare-associated infection, Global health, Survey

Quality healthcare should be available to everyone. The World Health Organization’s (WHO) concept of Universal Health Coverage (UHC), [1] embodies the urgent need for access to healthcare for all people around the world. In addition to access, the concept of UHC incorporates the critical element of the necessary quality of delivered health care services. Infection prevention and control (IPC) with hand hygiene as the most effective measure, is a practical and evidence-based approach with demonstrated impact on quality of care and patient safety across all levels of the health system.

Each year, the WHO *SAVE LIVES: Clean Your Hands* campaign aims to bring people together in support of hand hygiene improvement globally on or around May 5th. [2] This year’s theme for global annual hand hygiene day reflects a strong focus on providing clean care equally protecting all patients and healt PLs raise to PO if the author response in Q2 means that the presentation of the fifth author givenName and familyName in s200 process was correct? hcare workers from infection and antimicrobial resistance transmission, across all countries, including in low-resource PLs raise to PO if the author response in Q2 means that the presentation of the fifth author givenName and familyName in s200 process was correct? settings.

WHO urges ministries of health, health facility leaders, IPC leaders, health workers, and patient advocacy groups to contribute to effective IPC action including hand hygiene as a cornerstone of quality in healthcare (Table 1). WHO invites all healthcare facilities to join the 2019 WHO Global Survey on IPC and Hand Hygiene by using two validated assessment tools; one for evaluating the core components of IPC programmes and the other for a deep dive in hand hygiene activities (https://www.who.int/infection-prevention/campaigns/ipc-global-survey-2019/en/).

**Table 1:** May 5, 2019, World Health Organization *SAVE LIVES: Clean Your Hands* campaign calls to action

Campaign participants	Call to action
Health workers	“Champion clean care – it’s in your hands.”
IPC^a^ leaders	“Monitor infection prevention and control standards – take action and improve practices.”
Health facility leaders	“Is your facility up to WHO infection control and hand hygiene standards? Take part in the WHO survey 2019 and take action!”
Ministries of health	“Does your country meet infection prevention and control standards? Monitor and act to achieve quality universal health coverage.”
Patient advocacy groups	“Ask for clean care – it’s your right.”

^a^*IPC*, infection prevention and control

On a facility level, the use of these tools gives institutions a clear understanding of the strengths and weaknesses of their IPC and hand hygiene programmes, and provides concrete actions to address existing gaps. These tools allow institutions to improve their IPC practices and policies in a concrete and measurable way, at their own speed and in their own context. The surveys are anonymous, and global results will be made available only using aggregated data. This means that facilities and ministries of health can commit fully to working on improving IPC and patient safety without fear of scrutiny or possible negative repercussions.

Globally, this survey will allow WHO to provide a situational analysis on the level of progress of current IPC and hand hygiene activities around the world and inform future efforts and resource use for IPC capacity building and improvement. Global Surveys using the Hand Hygiene Self-Assessment Framework were already conducted in 2011 and 2015, [3–5] making this year’s survey even more crucial for tracking the implementation of hand hygiene and IPC on a global scale (Fig. 1).

**Fig. 1:**
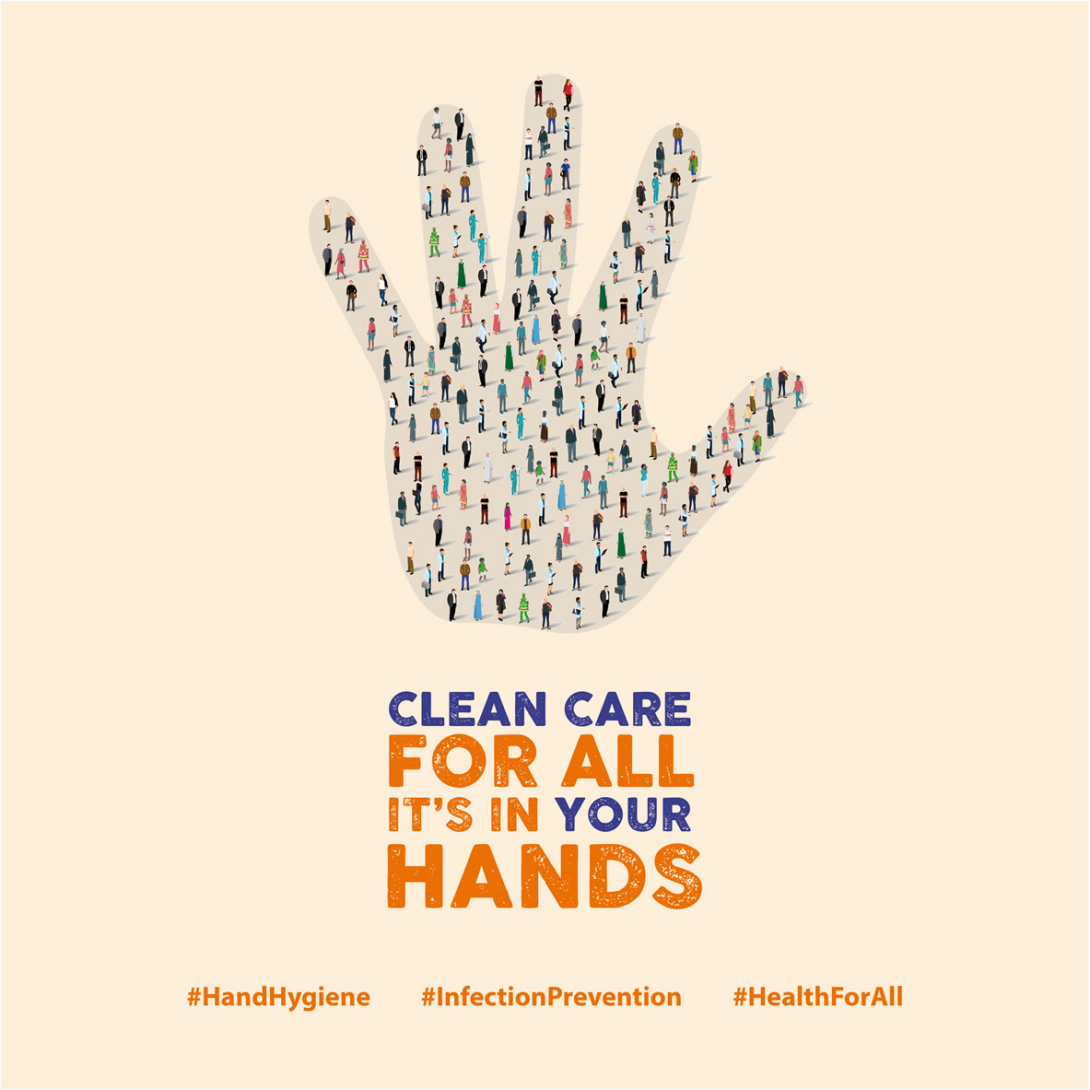
May 5, 2019: *“Clean care for all – it’s in your hands”*!. The May 5, 2019, World Health Organization *SAVE LIVES: Clean Your Hands* campaign slogan and main promotional image (2019 hashtags: #HandHygiene #InfectionPrevention #HealthForAll). Campaign participants are invited to submit photos or selfies of them holding a board with the slogan and hashtags at www.CleanHandsSaveLives.org

Each improvement in IPC contributes toward quality UHC. “Clean care for all – it’s in your hands”!

## Abbreviations

IPC: infection prevention and control
UHC: universal health coverage
WHO: World Health Organization
